# Six in ten female youths in low-income East African countries had problems in accessing health care: a multilevel analysis of recent demographic and health surveys from 2016–2021

**DOI:** 10.1186/s12913-024-10934-z

**Published:** 2024-04-26

**Authors:** Misganaw Guadie Tiruneh, Eneyew Talie Fenta, Destaw Endeshaw, Habitu Birhan Eshetu, Ousman Adal, Abiyu Abadi Tareke, Natnael Kebede, Amare Mebrat Delie, Eyob Ketema Bogale, Tadele Fentabel Anagaw

**Affiliations:** 1 Department of Health Systems and Policy, Institute of Public Health, College Medicine and Health Sciences, University of Gondar, Gondar, P.O. Box: 196, Ethiopia; 2 Department of Public Health, College of Medicine and Health Sciences, Injibara University, Injibara, Ethiopia; 3 Department of Adult Health Nursing, School of Health Sciences, College of Medicine and Health Sciences, Bahir Dar University, Bahir Dar, Ethiopia; 4 Department of Health Promotion and Health Behaviour, Institute of Public Health, College of Medicine and Health Sciences, University of Gondar, Gondar, Ethiopia; 5 Department of Emergency and Critical Care Nursing, College of Medicine and Health Sciences, Bahir Dar University, Bahir Dar, Ethiopia; 6 Amref Health Africa in Ethiopia, SLL project COVID-19/EPI technical assistant at West Gondar Zonal Health Department, Gondar, Ethiopia; 7 Department of Health Promotion, School of Public Health, College of Medicine and Health Sciences, Wollo University, Dessie, Ethiopia; 8 Department of Health Promotion and Behavioral Sciences, School of Public Health, College of Medicine and Health Sciences, Bahir Dar University, Bahir Dar, Ethiopia

**Keywords:** Access, Healthcare, East Africa, Youths

## Abstract

**Background:**

Access to health care services is a basic human right, and an individual’s health and overall quality of life may suffer as a result of barriers to accessing health services. Access to comprehensive and quality health care is fundamental for promoting and maintaining health, preventing and treating diseases, and reducing premature deaths. However, only half of the African population has access to modern health services. Therefore, this study aimed to assess the health care access and associated factors among female youths in low-income East African countries.

**Methods:**

This study used secondary data from 2016 to 2021 demographic and health surveys of 7 low-income East African countries. A total weighted sample of 51,064 youths was included. A multilevel binary logistic regression was employed to identify the associated factors of access to health care since the data has a hierarchical structure. Adjusted Odds Ratio (AOR) with a 95% confidence interval (CI) at a p-value less than 0.05 was used to measure the association of variables whereas Intra-class correlation coefficient (ICC), Median Odds Ratio (MOR), and proportional change in variance (PCV) were used to measure random effects.

**Result:**

The overall magnitude of access to healthcare among female youths in low-income East African countries was 38.84% (95% CI: 38.41, 39.26). Youth’s educational level, rich wealth status, media exposure, and community level education were the positive while higher youth’s age and rural residence were the negative predictors of access to healthcare among female youths. Besides, living in different countries compared to Burundi was also an associated factor for accessing healthcare in low-income East African countries.

**Conclusion:**

About six in ten female youths were not accessing health care in low-income East African countries. Therefore, to increase healthcare access, health managers and policymakers needed to develop strategies to improve the poor household wealth index, and redistribution of health services for rural residents. The decision-makers and program planners should also work on increasing access to education and media exposure for youths. Further research including health system and quality of service-related factors for accessing healthcare should also be considered by researchers.

## Background

According to the World Health Organization (WHO) definition, youth is an individual who is between the ages of 15 and 24 years [1]. Youths are the global future leaders who need essential and comprehensive health care [2, 3]. Youths need health information, including age-appropriate comprehensive sexuality education; opportunities to develop life skills; and health services that are acceptable, equitable, appropriate, and effective in safe and supportive environments to grow and develop with good health [4]. Over 1.5 million adolescents and young adults aged 10–24 years have died in 2021 due to preventable and treatable causes, globally. Thus, access to healthcare is essential to improve health and reduce the death of youths [3, 5, 6].

Access to health care services is a basic human right, and barriers to accessing health services may have a detrimental impact on an individual’s health, and overall quality of life [7, 8]. The world health community is setting an ambitious target of Universal Health Coverage (UHC) by 2030, whereas improvements to health services coverage have stagnated since 2015 [9]. Particularly, Sustainable Development Goal-3 (SDG-3) targets 3.8 and 3.7 emphasize Universal Health Coverage (UHC) and access to sexual and reproductive healthcare services, including family planning information and education, and the integration of reproductive healthcare into national strategies and programs by 2030 [9, 10]. Globally, about half of the world’s population can’t obtain essential health services [5]. Similarly, only half of the African population has access to modern health services [5]. Unfortunately, access to health care services is a major issue in low-income countries [5, 8, 9]. Besides, there is a huge gap exists in the availability and accessibility of services in Sub-Saharan Africa and Southern Asia [5, 11].

Access to comprehensive and quality health care is fundamental for promoting and maintaining health, preventing and treating diseases, reducing avoidable disabilities and premature deaths, and achieving health equity for all women [12]. Youths experience many barriers to health care, including financial, legal/structural (policy requires parental/partner consent, distance from health facility, cost of services/transportation), and societal barriers (restrictive norms and stigma) [6, 13–18].

Previous studies reported factors associated with access to health care including socio-demographic and economic determinants such as age [16, 19–23], residence [20, 24–26], educational level [15, 20, 22, 24, 26, 27], husband’s educational level [26, 27], marital status [19, 20, 24, 28], wealth index [19, 20, 22–27], occupation [24], wanted pregnancy [25, 26], having living children [24], women’s household decision-making autonomy [16, 29], covered by health insurance [19, 23], and media exposure [19, 30].

Different studies related to healthcare access among youths have been conducted at the country level [31–34]. However, there is no evidence of access to healthcare among female youths in low-income East African countries. Besides, to achieve SDG 3 identifying the factors of access to healthcare is essential to enhance universal healthcare access that assures the health needs of female youths. Therefore, this study aimed to generate evidence of healthcare access among female youths by including data from seven low-income East African countries from 2016 to 2021. The findings of the study will help in the decision-making and problem-solving process to improve healthcare access and to formulate an intervention strategy to address issues of poor youth health status and outcomes.

## Methods

### Study design and setting

This study was conducted in seven East African countries using the Demographic and Health Survey (DHS) data collected between 2016 and 2021 to assess the health care access and associated factors among female youths (15–24 years old) in low-income East African countries. These countries are Burundi, Ethiopia, Madagascar, Malawi, Rwanda, Uganda and Zambia. According to World Bank income classifications Comoros, Djibouti, Kenya, Mauritius, Seychelles, Tanzania, and Zimbabwe were out of the low-income countries. Mayotte, Reunion, South Sudan, and Somalia were not included due to the lack of a DHS dataset. Furthermore, Sudan and Eretria were also excluded due to the long period since their last standard DHS. The DHS used a community-based cross-sectional study design to collect the data at the national level [35].

### Data source and study population

The analysis was based on the secondary data of the most recent DHS of low-income East African countries. The DHS program collects standard and comparable data in low and middle-income economic class countries. The program designs the same manual, variable name, code, value level, and procedure in more than 90 countries across the world [26, 35]. The survey used a two-stage stratified sampling technique every five years. In the first stage, enumeration area clusters were selected by proportional sample size method. Then, a fixed number of households per cluster was selected by equal probability systematic sampling following the list of households [35]. The DHS data were collected using face-to-face interviews of reproductive-age women from 15 to 49 years on variety of issues related to population, health, nutrition tracking, and evaluation assessment measures. Detailed survey methodology and sampling methods used in gathering the data are available [35]. All female youths aged 15–24 years in low-income East African countries were included in this study. Before analysis, weighting was done to get a representative sample by dividing the individual weight for women (v005) by 1,000,000 to estimate the number of cases [35]. The total weighted sample size for this study was 51,064: Pooled Demographic and Health Survey (DHS) data were obtained from 7 low-income East African countries: Burundi (2016/17; 7,103), Ethiopia (2016; 6,143), Madagascar (2021; 7,906), Malawi (2015/16; 10,421), Rwanda (2019/20; 5,672), Uganda (2016; 8,086), and Zambia (2018; 5,733).

### Variables of the study

#### Outcome variable

The dependent variable of this study was access to health care. To ascertain the outcome variable we created a composite variable by using the DHS questions. The questions included: getting permission to go, getting the money needed for treatment, distance to healthcare facility, and not wanting to go alone [35, 36]. The responses to the questions were categorized as “big problem” and “not a big problem”. If youths face at least one problem, the youths have a big problem and are coded as “0”, and youths with not a big problem are coded as “1” [25, 26, 35].

#### Independent variables

The independent variables were considered because of their clinical and statistical relationship with healthcare access in the previous studies [6, 13, 15, 22, 24–26, 31, 36, 37]. This study identified individual and community level factors; the individual level variables include; age, educational level, husband’s educational level, marital status, wealth index, respondent occupation, current status of pregnancy, wanted pregnancy, having living children, decision-making autonomy for health care, covered by health insurance and media exposure. In addition, residence, countries, community-level media exposure, community-level poverty, and community-level education were considered under community-level factors. The community-level education, community-level poverty, and community-level media exposure were generated by aggregating the individual level variables at the cluster level and categorizing them as low if the proportion is < 50%, and high if the proportion is ≥ 50% based on the median value [38].

##### Respondent occupation

Youths occupation status was classified as “working” and “not working” by merging those youths with different occupation types as “working” and individuals without work “not working”.

##### Wealth status

the variable wealth index was re-categorized as “Poor”, “Middle”, and “Rich” by merging poorest and poorer as “poor” and richest with richer as “rich”.

##### Decision-making autonomy

was measured by the decision to use health care and it was categorized as “not autonomous”=0 (for youths who reported that the decision regarding their use of healthcare was made primarily by their partner alone or by someone else) and “autonomous” = 1 (for youths who reported that the decision regarding their use of healthcare was primarily made by the respondent alone and/or jointly with their partners) [39, 40].

##### Media exposure

was generated from the frequency of listening to the radio, watching television, and reading a newspaper or magazine. Respondents who never listened to the radio, read newspapers or watched television were considered as having no exposure to mass media, and otherwise exposed to mass media [41].

### Modeling approaches

We pooled data from seven low-income East African countries after extracting variables based on the literature. The extracted data from the included countries were weighted using sampling weight (v005) to obtain a valid statistical estimation. Data were coded, cleaned, and analyzed by using Stata V.17 software. Variables with p-value < 0.2 in the bi-variable multilevel logistic regression were fitted into the multivariable model. Adjusted Odds Ratio (AOR) with a 95% CI in the multivariable model was used to declare a statistically significant association with the female youth’s access to healthcare.

A multilevel logistic regression model was used to identify the association between the individual and community-level factors of access to health care among female youths of low-income East African countries. Due to the hierarchical nature of DHS data (individuals are nested into the communities), which violates the equal variance and independent observations assumptions of a traditional logistic regression model, it is advised to use multilevel analyses to take into account such data [42, 43]. A multilevel model also allows tracking changes in variance and the Likelihood ratio (LR) test can be used to determine whether the standard logistic regression or the multilevel model fits the data best, in this study the LR test was significant (*p* < 0.05). As a result, the multilevel model was preferred over the standard logistic regression model. This indicates that if we use a standard logistic regression model in the presence of significant LR, the result becomes biased and ends up with a wrong conclusion [44].

Four models were fitted to identify the associated factors of access to health care among female youths in low-income East African countries. The null model (model 0) shows the variations in access to health care in the absence of any explanatory variables, model I (a model that includes only individual-level factors), model II (a model that includes only community-level factors), and model III (a model includes both individual and community level factors). The variation among clusters was assessed by Intra-class Correlation Coefficient (ICC), Proportional Change in Variances (PCV), and Median Odds ratio (MOR) [45, 46].

## Result

### Individual and community-level characteristics of participants

A total of 51,064 female youths were included in this study. Out of the study participants, 53.29% were between the ages of 15–19 years, and the median age with the interquartile range of participants was 19 (IQR: 17–22 years). About two-thirds of the participants were single and the majority, 87.33% were not covered by health insurance. Half of the participants attended primary education. Regarding residence, 76.15% of them were from rural areas. Half of the participants were from communities with a low proportion of community-level media exposure and community-level education. The highest number of youth participants was from Malawi, 10,421 (20.41%), and the lowest number of study participants was from Rwanda 5,672 (11.11%) (Table 1).

**Table 1:** Individual and community level characteristics of youths in low-income East Africa (*n* = 51,064)

Variable	Category	Weighted frequency	Percent
Age	15–19 years	27,210	53.29
	20–24 years	23,854	46.71
Sex of household head	Male	36,760	71.99
	Female	14,304	28.01
Respondents educational level	No formal education	4,002	7.84
	Primary education	25,882	50.69
	Secondary education	19,444	38.08
	Higher	1,736	3.40
Respondents occupation	Currently working	21,932	42.98
	Currently not working	29,132	57.02
Marital status	Single	32,872	64.37
	Married	18,192	35.63
Husband’s educational level	No formal education	2,308	12.69
	Primary education	8,772	48.22
	Secondary	5,929	32.59
	Higher	841	4.62
	Don’t know	342	1.88
Wealth index	Poor	18,454	36.14
	Middle	9,650	18.90
	Rich	22,960	44.96
Currently pregnant	Yes	4,261	8.35
	No	46,803	91.65
Wanted pregnancy	Yes	4,178	98.04
	No	83	1.96
Having living children	Yes	20,043	39.25
	No	31,021	60.75
Family size	1–5	28,008	54.85
	> 5	23,056	45.15
Decision-making autonomy for healthcare	Autonomous	13,120	72.12
	Not autonomous	5,072	27.88
Beating is justified if the wife goes without telling husband	Yes	5,027	27.63
	No	13,165	72.37
Covered by health insurance	Yes	6,467	12.67
	No	44,597	87.33
Media exposed	Yes	32,791	64.22
	No	18,273	35.78
Residence	Urban	12,180	23.85
	Rural	38,884	76.15
Countries	Burundi	7,103	13.91
	Ethiopia	6143	12.03
	Madagascar	7,906	15.48
	Malawi	10,421	20.41
	Rwanda	5,672	11.11
	Uganda	8,086	15.83
	Zambia	5,733	11.23
Community level poverty	Low	26,181	51.27
	High	24,883	48.73
Community level education	Low	25,554	50.04
	high	25,510	49.96
Community-level media exposure	Low	25,243	49.43
	High	25,821	50.57

**Fig. 1:**
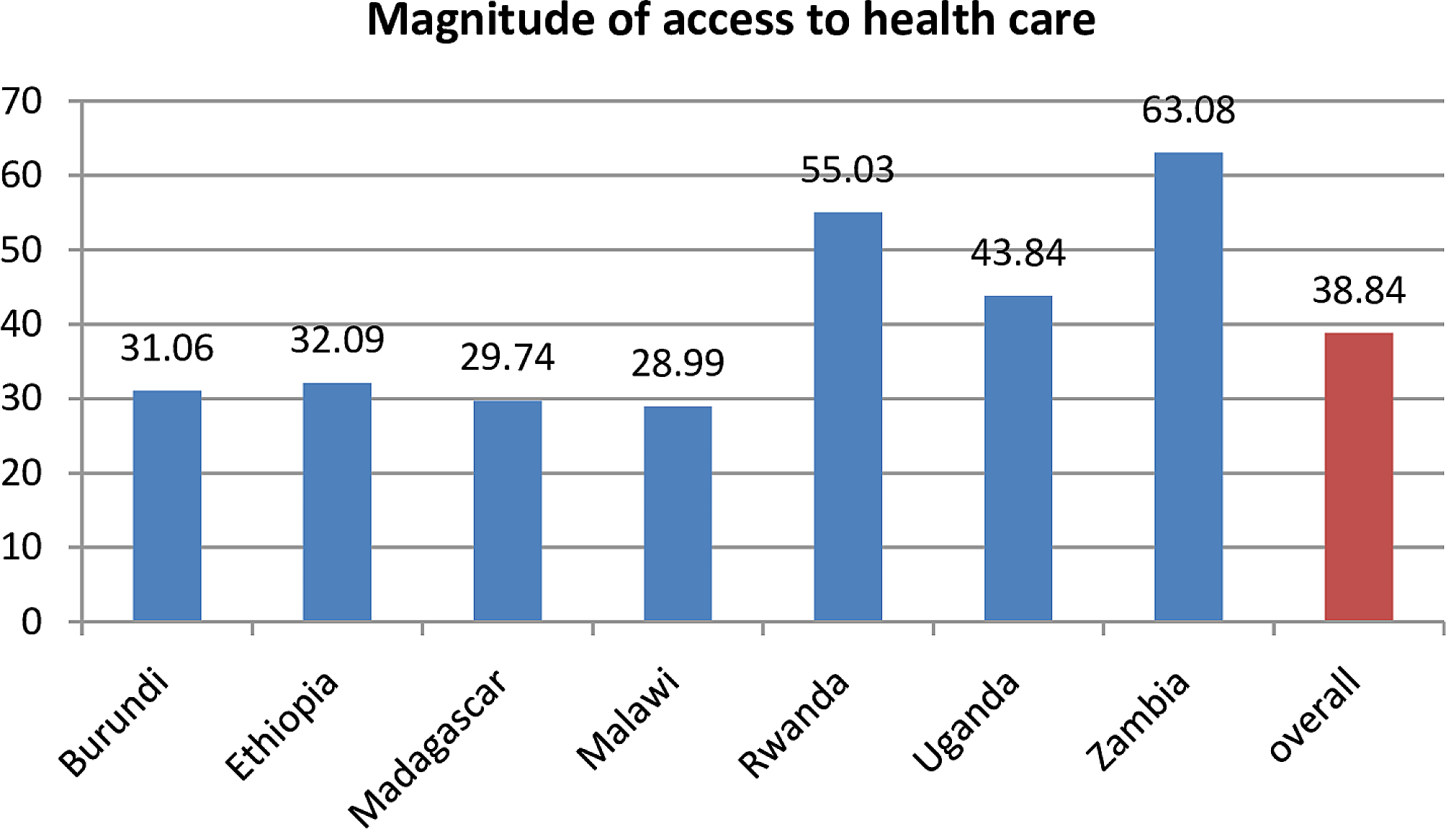
Magnitude of access to health care among female youths in low-income countries of East Africa

The overall magnitude of access to health care among female youths in low-income East African countries was 38.84% (95% CI: 38.41, 39.26). The highest magnitude of access to health care among female youths was in Zambia (63.08%) and the lowest magnitude of access to health care was in Malawi (28.98%) (Fig. 1).

### Random effect and model fitness

The null model in the random effects revealed statistically significant differences in the likelihood of access to health care with a variance of 59%. Additionally, the Intra-class Correlation Coefficient (ICC) in the null model 7.08% of the total variability of access to health care among female youths accounted for differences among clusters. The ICC refers to the ratio of cluster variance to total variance, and it indicates the proportion of the total variance in the outcome variable that is accounted for at the cluster level. Furthermore, the evidence of variation in access to healthcare among female youths was described by the Median Odds Ratio (MOR). Considering clusters as a random variable, the MOR indicates the median value of the odds ratio between the area at the highest risk and the area at the lowest risk when randomly picking out two different clusters. Accordingly, the odds of access to healthcare were 1.13 times higher among youths with a higher cluster of access to healthcare than youths with a lower cluster of access to healthcare. Regarding model comparison, the deviance (-2loglikilihood) was used to estimate the model fitness of the final adjusted model compared to the preceding models. A model with the lowest value of deviance is better; accordingly, model III was selected as the final model since it has the lowest deviance (4105.9) (Table 2).

### Factors associated with access to health care among female youths in low-income East African countries

In the final model, after we adjusted the individual and community level factors youth age, youth educational level, wealth index, and media exposure were the associated factors of access to healthcare among female youths from the individual level factors. In community-level factors, place of residence, community-level education, and country were statistically significant factors to access to health care among female youths in low-income East African countries.

Accordingly, the odds of accessing healthcare among aged 20–24 years were decreased by 25% (AOR = 0.75: 95% CI: 0.61, 0.92) compared to 15–19 years youths. The likelihood of accessing health care among female youths who had primary and secondary education levels was increased by 40% (AOR = 1.40; 95% CI: 1.04, 1.90), and 73% (AOR = 1.73; 95% CI: 1.23, 2.46) as compared to illiterate youths, respectively. Additionally, the odds of accessing healthcare were 1.35 times (AOR = 1.35; 95% CI: 1.07, 1.72) higher among youths from rich household wealth status as compared to youths from poor households. Concerning media exposure, the odds of accessing healthcare among youths who had media exposure was 1.35 times (AOR = 1.35; 95% CI: 1.12, 1.63) higher than their counterparts.

Rural resident youths were 53% (AOR = 0.47; 95% CI: 0.36, 0.61) less likely to access healthcare than urban resident youths. This study also found that the odds of accessing healthcare of youths from Madagascar and Malawi decreased by 52% (AOR = 0.48; 95% CI: 0.34, 0.68), and 58% (AOR = 0.42; 95% CI: 0.30, 0.59), as compared to youths from Burundi, respectively. In addition, the odds of accessing healthcare were 1.66 times (AOR = 1.66; 95% CI: 1.13, 2.44) higher among youths from Zambia as compared to youths from Burundi. Furthermore, accessing healthcare was 1.26 times (AOR = 1.26: 95% CI: 1.02, 1.55) high odds among youths from a high proportion of community-level education as compared to their counterparts (Table 2).

**Table 2:** Multilevel analysis of factors associated with access to health care among female youths (*n* = 51,064)

Variables	Access to healthcare		Null model	Model I AOR (95%CI)	Model II AOR (95%CI)	Model III AOR (95%CI)
	Not a big problem (%)	Big problem (%)				
**Age**						
15–19 years	10,606 (38.98)	16,604 (61.02)		1		1
20–24 years	9,226 (38.68)	14,628 (61.32)		0.80 (0.66, 0.98)		0.75 (0.61, 0.92)**
**Sex of household head**						
Male	14,328 (38.98)	22,432 (61.02)		1		1
Female	5,504 (38.48)	8,800 (61.52)		0.78 (0.61, 1.00)		0.79(0.62, 1.02)
**Respondents educational level**						
No formal education	930 (23.22)	3,072 (76.78)		1		1
Primary education	8,713 (33.66)	17,169 (66.34)		1.48 (1.11, 1.98)		1.40 (1.04, 1.90)*
Secondary education	9,144 (47.03)	10,300 (52.97)		2.00 (1.44, 2.78)		1.73(1.23, 2.46)**
Higher	1,046 (60.26)	690 (39.74)		2.00 (1.01, 3.96)		1.20 (0.59, 2.44)
**Respondents occupation**						
Currently working	9,486 (43.25)	12,446 (56.75)		0.95 (0.77, 1.10)		1.03 (0.85, 1.24)
Currently not working	10,357 (35.55)	18,756 (64.45)		1		1
**Husband’s educational level**						
No formal education	566 (24.52)	1,742 (75.48)		1		1
Primary education	2,716 (30.96)	6,056 (69.04)		1.00 (0.75, 1.34)		0.98 (0.73, 1.32)
Secondary	2,583 (43.57)	3,346 (56.43)		1.38 (1.00, 1.90)		1.26 (0.90, 1.76)
Higher	449 (53.34)	392 (46.66)		1.30 (0.77, 2.18)		1.14 (0.67, 1.94)
Don’t know	150 (43.91)	192 (56.09)		1.63 (0.91, 2.92)		1.39 (0.76, 2.55)
**Wealth index**						
Poor	5,036 (27.29)	13,418 (72.71)		1		
Middle	3,389 (35.12)	6,261 (64.88)		1.17 (0.94, 1.46)		1.14 (0.91, 1.43)
Rich	11,406 (49.68)	11,554 (50.32)		1.60 (1.30, 1.97)		1.35 (1.07, 1.72)*
**Wanted pregnancy**						
Yes	1576 (37.74)	2601 (62.26)		0.86 (0.37, 1.97)		0.88 (0.36, 2.12)
No	23 (27.52)	60 (72.48)		1		1
**Having living children**						
Yes	7174 (35.80)	12,868 (64.20)		1.21 (1.01, 1.46)		1.15 (0.95, 1.39)
No	12,657 (40.80)	18,364 (59.2)		1		1
**Family size**						
1–5	10,437 (37.27)	17,571 (62.73)		1		1
>5	9,395 (40.75)	13,661 (59.25)		0.86 (0.69, 1.08)		0.83 (0.66, 1.05)
**Decision making autonomy for health care**						
Autonomous	4,807 (36.63)	8,313 (63.37)		1.19 (1.00, 1.42)		1.19 (0.99, 1.43)
Not autonomous	1,657 (32.68)	3,414 (67.32)		1		1
**Beating justified if wife goes without telling husband**						
Yes	1727 (34.36)	3300 (65.64)		1.02(0.86, 1.23)		0.88 (0.73, 1.06)
No	8096 (61.5)	5,069 (38.5)		1		1
**Covered by health insurance**						
Yes	3625 (56.06)	2842 (43.94)		1.64 (1.20, 2.25)		1.35 (0.84, 2.18)
No	16,207 (36.34)	28,390 (63.66)		1		1
**Media exposed**						
Yes	14,530 (44.31)	18,261 (55.69)		1.42 (1.19, 1.70)		1.35 (1.12, 1.63)**
No	5302 (29.02)	12,971 (70.98)		1		1
**Residence**						
Urban	6846 (56.21)	5334 (43.79)			1	1
Rural	12,986 (33.40)	25,898 (66.60)			0.41 (0.39, 0.43)	0.47 (0.36, 0.61)***
**Countries**						
Burundi	2206 (31.06)	4897 (68.94)			1	1
Ethiopia	1972 (32.09)	4171 (67.91)			1.03 (0.95, 1.12)	0.74 (0.51, 1.09)
Madagascar	2351 (29.74)	5555 (70.26)			0.85 (0.79, 0.92)	0.48 (0.34, 0.68)***
Malawi	3020 (28.99)	7401 (71.01)			0.90 (0.84, 0.96)	0.42 (0.30, 0.59)***
Rwanda	3121 (55.03)	2551 (44.97)			2.75 (2.55, 2.97)	0.95 (0.54, 1.66)
Uganda	3545 (43.84)	4541 (56.16)			1.60 (1.49, 1.72)	1.01 (0.73, 1.40)
Zambia	3617 (63.08)	2116 (36.92)			3.16 (2.92, 3.42)	1.66 (1.13, 2.44)*
**Community level poverty**						
Low	11,120 (42.47)	15,061 (57.53)			1	1
High	8712 (35.01)	16,171 (64.99)			0.86 (0.79, 0.93)	0.83 (0.66, 1.04)
**Community level education**						
Low	9441 (36.95)	16,113 (63.05)			1	1
High	10,391 (40.73)	15,119 (59.27)			1.09 (1.02, 1.18)	1.26 (1.02, 1.55)*
**Community-level media exposure**						
Low	8,968 (35.52)	16,275 (64.48)			1	1
High	10,864 (42.08)	14,957 (57.92)			1.05 (0.96, 1.14)	0.87 (0.70, 1.09)
**Measures of variations**						
Variance			0.59	0.25	0.56	0.19
ICC (%)			7.08%	15.3%	14.6%	5.4%
MOR			1.98	1.29	1.93	1.13
PCV			Ref.	57.6%	5.08%	67.8%
**Deviance (-2logliklihood)**			66,835.6	4,271.8	62,680.3	4,105.9

The null model contains no explanatory variables; Model I includes individual-level variables only; Model II includes community-level factors only; Model III includes both individual and community-level factors, AOR: Adjusted Odds Ratio, CI: Confidence Internal, ICC: Intra-class Correlation Coefficient, MOR: Median Odds Ratio, PCV: Proportional Change in Variance

*: p-value less 0.05; **: p-value less than 0.01; ***: p-value less than 0.001

## Discussion

The study aimed to assess the magnitude of access to healthcare and associated factors among female youths in low-income East African countries. This study found that the overall magnitude of access to healthcare among female youths in low-income East African countries was 38.84%. Regarding the determinants of access to healthcare; youth’s age, youth educational level, wealth index and media exposure from the individual level, and place of residence, community-level education, and country from the community level factors were statistically significant predictors of access to healthcare among female youths in low-income East African countries.

The coverage of access to healthcare among female youths in low-income East African countries was 38.84% (95% CI: 38.41, 39.26), which is comparable with a study done in SSA 38.5% [19]. However, it is higher than a study conducted in Ethiopia [20, 22, 24] and Tanzania [6]. The discrepancy might be attributed to the variation in the study setting and the study population, where the previous studies were conducted in a single country with the inclusion of all reproductive-age women, whereas this study included only female youths in low-income East African countries.

The current study finding showed a lower magnitude of access to healthcare than study reports in SSA [26], Ethiopia [31], Vermont [23], Gambia [36], and Myanmar [29]. The difference in the study population might be the reason for the variation. Moreover, it is lower than the study done in East African countries [25]. The variation might be attributed to the study setting and study population differences. Even though both of the studies were conducted in the same study setting in East Africa, the previous study was done on reproductive-age women of all East African countries, while this study was conducted on female youths of low-income East African countries. In this regard, the previous study done in East Africa had a large sample size with the inclusion of all reproductive-age women, which may in turn increase the magnitude of healthcare access.

This study revealed that the odds of accessing healthcare were lower among youths aged 20–24 years as compared to youths aged 15–19 years. This finding is in line with a study conducted in Ethiopia [31], SSA [19], and Tanzania [6]. The possible justification might be that adolescents aged 15–19 years may be better cared for by their families than young of aged 20–24 years, which increases their access to health care.

Consistent with previous studies conducted in Ethiopia [15, 20, 22, 24, 25, 31, 47], Tanzania [6], Myanmar [29], and SSA [6, 19], youths who attended primary and secondary schools had a higher likelihood of accessing health care as compared to illiterates. This could be explained by education may improve awareness and increase health-seeking behavior [20], or it could be due to education being the major factor of higher employment opportunities which may in turn increase accessibility to healthcare services [22, 31]. Furthermore, education enhances the freedom and decision-making capacity of women in matters related to their health and reproductive health services [22]. This implies that the government should establish different educational programs that address the literacy gaps of female youths to empower them.

This study also found that the odds of accessing healthcare were high among rich households as compared to poor household wealth status. This result is consistent with studies conducted in Ethiopia [22, 24, 25, 31, 47], Gambia [36], Myanmar [29], Tanzania [6], and SSA [19, 26]. The possible explanation might be due to a rich wealth index may reduce the difficulties of obtaining money to access health care [20]. Additionally, money is the basis for covering basic needs and health-related costs. The finding implies that there is a need to develop strategies and initiatives that help to improve the low socio-economic status and increase the health care access for female youths.

In this study, media exposure is another determinant of healthcare access among female youths in low-income East African countries. Media-exposed female youths had higher access to healthcare than their counterparts. This study finding is supported by a study done in Ethiopia [31]. This could be due to the fact that media can be useful for the dissemination of health information and healthcare, which may improve knowledge, attitudes, and practices related to health service utilization [31], or it might be because media can assist health professionals in broadening their audience, which doesn’t require too many human resources and reach to large individuals including rural areas. This implies that the government should work with the media to deliver health information to improve healthcare access.

Additionally, access to healthcare among rural residents was lower as compared to urban residents. Other studies in SSA [19, 26], Ethiopia [24, 25, 31], Gambia [36], and Myanmar [29] also supported our findings. The possible reason might be that rural areas are associated with lower geographical accessibility of health facilities. In addition to geographical accessibility and economic problems, there are also socio-cultural issues related to lower male involvement and support for young women’s healthcare access [20]. This finding indicates that the government should build and expand infrastructure for those who reside in rural areas.

Community-level education was also found to be a significant predictor of access to healthcare. The likelihood of accessing healthcare among female youths from a high proportion of community-level education was high as compared to their counterparts. This study finding is in agreement with studies conducted in Ethiopia [22], and Benin [37]. The possible explanation could be due to the association of education with income level [22, 37]. Education is an essential element for increasing the health and overall wellness of persons. It aids in promoting and sustaining wholesome lifestyles and positive selections, thereby augmenting human development as a whole [37]. Furthermore, youths from Madagascar and Malawi had lower odds of accessing health care as compared to Burundi. Besides, Youths from Zambia had high odds of accessing health care as compared to Burundi. The possible reason might be due to the countries’ differences in terms of their health system, policies, government structure, and health institutions [25].

## Strengths and limitations

The major strength of this study was the use of a large sample size of representative data collected with standard and validated data collection tools. In addition, the use of multilevel modeling, a model which considers the hierarchical nature of the DHS data was also the strength of this study. However, this study is not free from limitations. The first limitation is due to the cross-sectional nature of the data the study can’t establish a causal relationship between the outcome variable and independent variables. Additionally, since we used secondary data health system and service quality-related factors could not addressed. Furthermore, we pooled DHS data from different countries collected in different year there might be changes in demographics, health policies, and other factors over time that could affect the comparability of the data.

## Conclusion

Only about four in ten female youths were accessing health care in low-income East African countries. Thus, to increase healthcare access, health managers and policymakers needed to develop strategies to improve the household wealth status for those with poor household wealth index and the redistribution of healthcare services for rural residents. The decision-makers and program planners should also work on media exposure and increase access to education for youths. Further research, including health systems and quality of service-related factors for accessing healthcare, with consideration of sample size and DHS survey year differences should also be considered by researchers.

## Data Availability

Data used in our study are publicly available upon request from the DHS program website. (https://dhsprogram.com/).

## Abbreviations

AOR: Adjusted Odds ratio
CI: Confidence Interval
DHS: Demographic and Health Survey
ICC: Intra-class Correlation Coefficient
IQR: Interquartile Range
LR: Likelihood Ratio
MOR: Median Odds Ratio
PCV: Proportional Change in Variance
SDG: Sustainable Development Goal SSA: Sub-Saharan Africa
UHC: Universal Health Coverage
